# The geographic penetration of the JACMP and the benefits of AAPM membership for clinical medical physicists worldwide

**DOI:** 10.1002/acm2.12096

**Published:** 2017-05-02

**Authors:** Michael D. Mills

An analysis of the countries of origin for JACMP submissions shows notable growth respecting nations along the Pacific Rim. Specifically, we are seeing more articles submitted from Australia, China, Indonesia, Japan, Malaysia, New Zealand, and South Korea. Indeed, submission from Pacific Rim nations (excluding the US and Canada) comprised over 30% of all JACMP submissions in 2016.

In informal discussions with representatives of our publisher Wiley, this regional growth is considered significant. In response, the JACMP Board of Editors has identified a number of new JACMP Associate Editors from Pacific Rim nations that hopefully will encourage a continuation of this growth. The goal of the JACMP is to reach all English‐speaking clinical medical physicists, worldwide, and the Pacific Rim is a primary constituency.

Wiley has noticed a sharp increase of interest and number of submissions for all of its open access journals in the last 2 years. This interest is universal, but especially remarkable along the Pacific Rim. The JACMP seems to be benefiting by being in the right place at the right time with a distinguished publisher with significant presence in the open access publishing paradigm shift.

All of this brings to mind the old paradox of the relationship between a scientific society and its publication offerings. What is it that creates the community? Is it the society? Or is it the publishing community of scientists, clinicians, editors, reviewers, and authors that generate the activity that adds meaning and life to the society and its structure. I recall that for a few years in the early 1970s, AAPM membership languished. Then with the establishment of the Medical Physics Journal, membership grew a factor of four in just a few years. The perception at the time was that in order to be successful in the profession, you had to have access to the Medical Physics Journal, and in order to get the Journal, you had to be a member of the AAPM. So what does this mean for the JACMP, when the open access publishing model gives its content away without cost?

I believe a very strong case can be made for clinical medical physicists outside the US who are publishing in the JACMP to become AAPM members. Please consider this list of benefits contained within this webpage: https://www.aapm.org/memb/default.asp and this table: https://www.aapm.org/memb/DuesBenefits.pdf.

Notable AAPM member benefits include the ICRU and NCRP publications, the AAPM Professional Practice Surveys, the Career Services and Bulletin, the AAPM Newsletter, Online Continuing Education, Professional Liability and other Insurance, and the Member Directory which allows you to network with all AAPM members. I have not yet mentioned the most significant benefit, which is access to the Medical Physics Journal, with its entire archive of classic medical physics articles. Access to the literature is critically important, but it is not everything. Clinical medical physicists also need access to education resources and professional survey and other information that will help build a profession within a nation or community.

You will find there are many cost‐effective memberships available to you, our international colleagues, including student, resident, junior, corresponding and international affiliate membership categories. One of these is likely a good fit for you. As a forty‐plus year member of the AAPM, I cannot recommend the value of membership more highly. I believe it is your best move to advance your career and lead your profession.

## Conflict of Interest

The authors have no relevant conflicts of interest to disclose.

